# Comparative analysis of perceptual evaluation, acoustic analysis and indirect laryngoscopy for vocal assessment of a population with vocal complaint

**DOI:** 10.1016/S1808-8694(15)31278-7

**Published:** 2015-10-20

**Authors:** Kátia Nemr, Ali Amar, Marcio Abrahão, Grazielle Capatto de Almeida Leite, Juliana Köhle, Alexandra de O. Santos, Luiz Artur Costa Correa

**Affiliations:** 1 Ph.D. in Social Psychology, USP. Responsible for the Service of Speech and Hearing Pathology, Hospital Heliópolis; 2 Master in Head and Neck Surgery, HOSPHEL. Head and Neck Surgeon, Hospital Heliópolis; 3 Full Professor, Head and Neck Surgery, UNIFESP. Head of the Discipline of Head and Neck Surgery, UNIFESP; 4 Master studies under course, Program of Post-graduation Studies in Speech and Hearing Pathology, PUC/SP, Specialist in Voice, Irmandade da Santa Casa de Misericórdia de São Paulo. Speech and Hearing Pathologist, Service of Speech and Hearing Pathology, Hospital Heliópolis; 5 Specialist in Voice, CEFAC/SP. Speech and Hearing Pathologist, Service of Speech and Hearing Pathology, Hospital Heliópolis; 6 Specialist in Oral Motricity, CEFAC/SP. Speech and Hearing Pathologist, Service of Speech and Hearing Pathology, Hospital Heliópolis; 7 Master studies in Health Sciences, Hospital Heliópolis. Head and Neck Surgeon, Hospital Heliópolis

**Keywords:** evaluation, voice, spectrum analysis, laryngoscopy, speech, language and hearing pathology

## Abstract

As a result of technology evolution and development, methods of voice evaluation have changed both in medical and speech and language pathology practice.

**Aim:**

To relate the results of perceptual evaluation, acoustic analysis and medical evaluation in the diagnosis of vocal and/or laryngeal affections of the population with vocal complaint.

**Study design:**

Clinical prospective.

**Material and Method:**

29 people that attended vocal health protection campaign were evaluated. They were submitted to perceptual evaluation (AFPA), acoustic analysis (AA), indirect laryngoscopy (LI) and telelaryngoscopy (TL).

**Results:**

Correlations between medical and speech language pathology evaluation methods were established, verifying possible statistical signification with the application of Fischer Exact Test. There were statistically significant results in the correlation between AFPA and LI, AFPA and TL, LI and TL.

**Conclusion:**

This research study conducted in a vocal health protection campaign presented correlations between speech language pathology evaluation and perceptual evaluation and clinical evaluation, as well as between vocal affection and/or laryngeal medical exams.

## INTRODUCTION

The larynx is an important muscle-cartilaginous muscle whose functions are sound production, breathing and sphincter action, normally affected in diseases that are manifested as dysphonia.

Throughout the years, vocal assessment has been a subject of constant improvement by Speech and Hearing Pathology and Otorhinolaryngology and Head and Neck Surgery. Currently, subjective and objective criteria are part of the convergence of all assessment criteria.

Among the examples of diagnostic tools used to define laryngeal diseases we can include indirect laryngoscopy (Garcia's mirror), telelaryngoscopy (rigid endoscopy) and nasofibrolaryngoscopy (flexible endoscopy).

As to vocal assessment, it includes perceptual voice assessment and computer acoustic analysis.

Perceptual voice assessment of vocal function started in the 19^th^ century by subjectively assessing the voice with the human ear as assessment instrument. This practice has been used to present to detect affections, trying to identify the balance between what is seen and heard in the subject of the analysis and interpretation of findings, in which we learn and understand the individual dynamics of each one with their own voice and communication characteristics^1^.

As a result of instrument evolution, acoustic analysis was devised, which is a way to objectively assess voice; the advantages of this method are increase in diagnostic precision, identification and documentation of short and long-term treatment efficacy and possibility of providing visual feedback to the patient^2^.

Despite the advantages of acoustic analysis, it does not provide diagnosis, but it works as a complement to vocal assessment, together with physiological findings from the physical examination conducted by the physician and perceptual vocal analysis^3^. It is important to highlight that acoustic analysis is a complementary test that does not replace clinical assessment conducted by the speech and voice therapist, but it rather serves as another resource to help assessment.

All previously referred assessment methods, be them conducted by the physician or voice therapist, seem to be complementary in understanding and defining the management of cases that have vocal affections, which means that interdisciplinary approach is essential for successful resolution of cases. However, in view of the growing demand for vocal health protection actions, such as awareness campaigns and vocal problem prevention efforts, the discussion on the most appropriate methods to efficiently support these actions becomes more necessary.

The purpose of the present study was to relate results of perceptual voice assessment, acoustic analysis and medical assessment with the diagnosis of vocal and/or laryngeal affections in subjects with vocal complaints seen in a vocal health protection campaign.

## MATERIAL AND METHOD

The vocal health protection campaign was organized by the Service of Speech and Hearing Therapy, Hospital Heliópolis, together with Head and Neck Surgery and Post-Graduation Course on Health Sciences – HOSPHEL, counting on a team of speech and voice therapists, physicians and oro-maxillo-facial surgeons for one-day activities in which they assessed 80 subjects.

Inclusion criteria:

a) all subjects had some vocal complaint.

Exclusion criteria:

a) all subjects that presented other non-vocal complaint, with or without previous diagnosis.
b) any subject that could not or refuse to undergo any of the assessment methods.

Out of a total of 49 subjects with vocal complaints, 20 were excluded for not having conducted the exams. The sample comprised 29 subjects, 20 women and 9 men, aged 12 to 70 years, mean age of 44 years. The most important complaints were: hoarseness in 10 subjects (34%); difficulty to produce voice (such as abnormalities, voice breaks or vocal loss) in 9 subjects (31%); sore throat in 4 subjects (14%); vocal fatigue in 3 subjects (10%); other complaints (throat irritation, choking, short of breath) in 3 subjects (10%).

The method was divided into 4 stages:

Stage 1 – Perceptual voice assessment (AFPA), based on scale RASAT^4^, ^5^. The protocol led us to pointing no vocal affection (when all assessed criteria were within the normal range); or presence of vocal affection, regardless of grade or type of affection. The assessment comprised a sample of vowel production, number counting (1 to 10) and connected speech.
Stage 2 – Acoustic record of the voice using acoustic analysis software (AA) GRAM 5. 1. 7 by specially trained speech therapist (spectrogram version 5. 1. 7. R. S. Horne – available at www.monumental.com/rshorne/gram.html) and vocal recording in the PC and in cassette tapes (Panasonic tape recorder model RN-302). Subjects were asked to produce prolonged vowel/a/and to count from 1 to 10. Vocal spectrum were later analyzed by two professionals from the speech and vocal therapy team of the center, who had been specifically trained in the software, and the final result was the consensus of both analyses, considering vocal related aspects (general regularity of tracing, quality of harmonic recording, presence of interruptions, modulations and bifurcations, number of harmonics, presence of noise among the harmonics, replacement of harmonics by noise)^6^. We considered spectrum with and without affection, regardless of the type or grade of affection. The examiners did not have access to each others' results of the assessment protocol nor to recorded voices during the procedure.
Stage 3 – Conduction of indirect laryngoscopy with Garcia's mirror (LI) by physicians of the Service of Head and Neck.
Stage 4 – Conduction of telelaryngoscopy (TL) by two surgeons of the team of head and neck surgery of Hospital Heliópolis. This exam was conducted in a different date from the others, with previous scheduling.

In the medical exams described in stages 3 and 4 we considered the following findings: no affection (in cases in which no affection or visible abnormality was observed in the larynx) or presence of affection (in cases in which we observed affection or visible abnormality in the larynx, regardless of being irritative, functional or structural).

Each subject received a Manual of Vocal Guidance given by a speech and voice therapist^7^ (Nemr, Carvalho, Köhle 2002) in addition to guidance compatible with their cases, and referral to other assessments and/or vocal therapy, if necessary.

We defined the correlations between the assessment methods described in stages 1, 2, 3 and 4. The statistical analysis used Fischer Exact Test, considering significant p value equal or below 0.05.

Project Nº 230 and the Free Informed Consent of this study were approved by the Research Ethics Committee of the Institution.

## RESULTS

This study gathered 29 subjects. AFPA was affected in 22 subjects whereas AA was detected in 17, and 14 of them were assessed. TL was abnormal in 19 subjects.

Among the 7 subjects with abnormal AFPA, 2 had abnormal TL whereas there were 12 subjects with normal AA, and 7 had abnormal TL.

AFPA results were in agreement with AA in 62% of the cases, but AFPA showed higher correlation with the TL findings; in 77% of the cases there were abnormalities in both (p = 0.03).

Table 1 shows the correlation between the three assessments conducted in absolute numbers.

**Table 1:** Relationship between AFPA, AA and TL

				TL		
				no affections	with affections	Total
**AFPA**	no affections	**AA**	no affections	4	0	4
			with affections	1	2	3
	with affections	**AA**	no affections	1	7	8
			with affections	4	10	14
			Total	10	19	29

Legend:

AFPA - perceptual voice analysis

AA - acoustic analysis

TL - telelaryngoscopy

Despite the presence of vocal symptoms, there was no affection identified in assessments conducted in 4 (13%) subjects. In other 4 cases, the abnormalities observed in AFPA and AA were not related with TL affection.

Among the 19 subjects that presented TL abnormalities, 15 (79%) had affections detected in LI.

## DISCUSSION

The importance of vocal dynamic global assessment with auditory and visual aspects reinforces the relevance of this assessment method in speech and vocal practice, given it also considers body expression of dysphonia in complementation of vocal findings.

We should emphasize that in the studied sample, all subjects presented vocal complaints and high level of agreement between AFPA and medical exams, which showed positive association between them.

AFPA presented divergence between each speech and vocal therapist that analyzed the voice of subjects owing to professional formation, experience and especially auditory training of each of them. Some authors refer that even among trained professionals conducting this assessment, it is observed that in comparison to the diagnosis, the perceptual assessment presents low discrimination skill and low reliability to define normal range or vocal abnormalities^8^. However, it is an important method and still today it is used in practice^9^. In the present study, AFPA proved to be a valuable method given the agreement between AFPA and TL was 76%.

Interobserver studies should be developed to assess the level of agreement between examiners, a topic developed in some medical areas^10^, ^11^, ^12^. In our area, there are some studies along these lines, especially on interobserver assessment with different levels of knowledge and professional experience, as well as professionals from different countries; however, the authors did not find high levels of disagreement, pointing to perceptual scale as an excellent clinical instrument^13^, ^14^.

We should consider that in view of vocal complaint and/or abnormalities in one of the assessment, the subject should be further investigated. We observed that out of the total of cases that presented TL affections, 10% had no repercussion in AFPA. In cases of initial neoplasm of supraglottis and hypopharynx, for example, there may not be signals of perceptual auditory abnormality. In such cases, isolated perceptual assessment, in view of vocal complaint, may underestimate the severity of a subject, who should be referred to medical assessment.

Conversely, in some cases, abnormalities observed in AFPA were not confirmed by LI (24%) or TL (17%). In such cases, we should not disregard the possibilities of minor structural alterations of the vocal folds that can be diagnosed by stroboscopy; even if not present in most specialized clinics, it provides important information about the vibration cycle or pattern (dynamic characteristics of vocal fold mucosa), and the value of fundamental frequency^15^, ^16^, ^17^. However, laryngostroboscopy may also have limitations such as the possibility of not detecting minor structural alterations in the exam^18^. About 30% of minor structural alterations are diagnosed only at the surgical act^15^.

The literature points to indirect laryngoscopy as a very useful method to initially detect diseases^5^, ^19^. Even though there may be difficulties to detect minimum alterations, LI allows the exclusion of severe diseases that may be present without vocal affections.

Since AA is a highly sensitive method, some affections were not confirmed by the other assessment methods. In a study conducted with a population without vocal complaints, there were affections to acoustic parameters in all cases that presented some variation from the normal range, emphasizing acoustic analysis as a sensitive method^20^. This assessment is a valuable instrument in speech and vocal practice, both as visual aid to the patient to follow up phonotherapy progression, and in scientific research studies and in complementing speech and voice assessment^2^, ^3^. Some authors state that AA does not replace perceptual assessment in clinical practice, being complementary methods^21^.

In 14% of the cases in which voice and speech abnormalities were detected but not confirmed in TL we may correlate it with the fact of they having being conducted on different days. If on the screening day the subject had a cold, for example, which had disappeared by the day of the telelaryngoscopy, there would definitely be differences in the results, a fact that should be taken into account in future studies.

In the literature, many studies discussed the efficacy or inefficiency of these diagnostic methods; however, it seems to be a consensus the need to associate one more method with more precise knowledge of vocal dynamic and laryngeal conditions, especially in cases of vocal complaint^22^, ^23^, ^24^. Therefore, the findings in the present study reinforce the idea that the association of more than one method supports vocal assessment, especially the association of perceptual assessment with indirect laryngoscopy or telelaryngoscopy.

## CONCLUSION

This study conducted in a vocal health protection campaign with the population reporting vocal complaints showed agreement between perceptual assessment and medical assessment in the diagnosis of vocal and/or laryngeal abnormalities. Acoustic analysis is a complementary method that may be useful in the assessment of subjects with vocal complaints.
